# Optimization of CuO_x_/Ga_2_O_3_ Heterojunction Diodes for High-Voltage Power Electronics

**DOI:** 10.3390/nano15020087

**Published:** 2025-01-08

**Authors:** Xiaohui Wang, Mujun Li, Minghao He, Honghao Lu, Chun-Zhang Chen, Yang Jiang, Kangyao Wen, Fangzhou Du, Yi Zhang, Chenkai Deng, Zilong Xiong, Haozhe Yu, Qing Wang, Hongyu Yu

**Affiliations:** 1 School of Microelectronics, Southern University of Science and Technology, Shenzhen 518055, China; 12231174@mail.sustech.edu.cn (X.W.); 12132453@mail.sustech.edu.cn (M.L.); hemh@u.nus.edu (M.H.); luhh@mail.sustech.edu.cn (H.L.); 11510044@mail.sustech.edu.cn (Y.J.); 22112020122@m.fudan.edu.cn (K.W.); 12232505@mail.sustech.edu.cn (F.D.); zhangyi97@connect.hku.hk (Y.Z.); 12149033@mail.sustech.edu.cn (C.D.); 12431347@mail.sustech.edu.cn (Z.X.); 12333329@mail.sustech.edu.cn (H.Y.); 2 Peng Cheng Laboratory, Shenzhen 518000, China; chenchzh@pcl.ac.cn; 3 Department of Electrical and Computer Engineering, National University of Singapore, Singapore 119077, Singapore; 4 Faculty of Engineering, The University of Hong Kong, Hong Kong 999077, China; 5 School of Microelectronics, Fudan University, Shanghai 200433, China; 6 Engineering Research Center of Integrated Circuits for Next-Generation Communications, Ministry of Education, Southern University of Science and Technology, Shenzhen 518055, China; 7 The Key Laboratory of the Third Generation Semiconductor, Southern University of Science and Technology, Shenzhen 518055, China

**Keywords:** power electronics, β-Ga_2_O_3_, heterojunction diodes, Cu_2_O, optimization

## Abstract

This study optimizes the CuO_x_/Ga_2_O_3_ heterojunction diodes (HJDs) by tailoring the structural parameters of CuO_x_ layers. The hole concentration in the sputtered CuO_x_ was precisely controlled by adjusting the Ar/O_2_ gas ratio. Experimental investigations and TCAD simulations were employed to systematically evaluate the impact of the CuO_x_ layer dimension and hole concentration on the electrical performance of HJDs. The results indicate that increasing the diameter dimension of the CuO_x_ layer or tuning the hole concentration to optimal values significantly enhances the breakdown voltage (V_B_) of single-layer HJDs by mitigating the electric field crowing effects. Additionally, a double-layer CuO_x_ structure (p^+^ CuO_x_/p^−^ CuO_x_) was designed and optimized to achieve an ideal balance between the V_B_ and specific on-resistance (R_on,sp_). This double-layer HJD demonstrated a high V_B_ of 2780 V and a low R_on,sp_ of 6.46 mΩ·cm^2^, further yielding a power figure of merit of 1.2 GW/cm^2^. These findings present a promising strategy for advancing the performance of Ga_2_O_3_ devices in power electronics applications.

## 1. Introduction

Beta-gallium oxide (β-Ga_2_O_3_) has emerged as a highly promising ultra-wide bandgap material with great potential in the field of power electronics. Due to its large bandgap of 4.8 eV, high critical electric field of 8 MV/cm, and high Baliga’s figure of merit, Ga_2_O_3_ is an exceptional candidate for high-voltage applications, outperforming materials like silicon (Si), gallium nitride (GaN), and silicon carbide (SiC) [1,2,3]. In addition, the availability of melt-grown substrate production also makes it more economically desirable [4,5]. Over the past decade, the high-performance Ga_2_O_3_ devices, such as Schottky barrier diodes (SBDs), have been successfully demonstrated [6,7]. However, the absence of p-type conductivity remains a significant obstacle to the development of Ga_2_O_3_ bipolar power electronics [8]. To address this challenge, the p-n heterojunction diodes (HJDs) that integrate n-type Ga_2_O_3_ with alternative p-type materials have been explored as a promising solution.

In this regard, various p-type oxide semiconductors, including cuprous oxide (Cu_2_O) [9], nickel oxide (NiO) [10,11], tin oxide (SnO) [12], and chromic oxide (Cr_2_O_3_) [13,14], have been investigated for developing Ga_2_O_3_ HJDs. Among these, Cu_2_O stands out due to its non-toxic, low-cost, and bandgap energy of 2.1 eV. Cu_2_O films are particularly promising as p-type active layers in heterostructures, benefiting from their high hole mobility [15]. It has been reported that the p-type behavior of Cu_2_O is attributed to the negatively charged copper vacancies or nitrogen doping [16]. To date, the p-Cu_2_O/n-Ga_2_O_3_ HJDs with a breakdown voltage (V_B_) of 1490 V and a specific on-resistance (R_on,sp_) of 8.2 mΩ·cm^2^ have been successfully demonstrated, firstly making Cu_2_O as a viable candidate for integration with Ga_2_O_3_ in power devices [9]. Subsequent studies have explored the electrical characteristics of Cu_2_O/Ga_2_O_3_ HJDs, including those prepared by sputtering Cu_2_O films at 600 °C, which achieved a V_B_ of 1015 V and an R_on,sp_ of 8.32 mΩ·cm^2^ [17]. Additionally, the ampere-class Cu_2_O/Ga_2_O_3_ trench heterojunction barrier Schottky diodes have shown potential in high-voltage, high-current applications, demonstrating a maximum current up to 3.5 A, a leakage current of 1.3 × 10^−3^ A/cm^2^, and a V_B_ of 986 V [18]. Whereas Cu_2_O/Ga_2_O_3_ HJDs still suffer from poor device performance and require more comprehensive guidelines for design optimization.

Previous studies have shown that the fabrication process of p-type layers plays a crucial role in determining the heterojunction quality and, consequently, the electrical performance of the HJDs. The design optimization of NiO/Ga_2_O_3_ HJDs has been extensively explored [19]. The hole concentration in NiO layers was controlled by adjusting the flow rate of argon/oxygen (Ar/O_2_). Additionally, the impact of NiO layer geometry on the HJDs’ performance was investigated. The improvements in device performance were achieved by either enlarging the diameter dimensions of the NiO layer or tuning the hole concentration to alleviate the electric field crowding effect [20]. However, a precise design strategy for the Cu_2_O/Ga_2_O_3_ heterojunctions is still unclear. Therefore, achieving further enhancements in the performance of Cu_2_O/Ga_2_O_3_ HJDs requires an elaborate and detailed optimization approach.

In this work, we present an optimization approach for the CuO_x_/Ga_2_O_3_ HJDs by adjusting the structural parameters of the CuO_x_ layer, including its hole concentration and geometric dimensions. A combination of experimental studies and technology computer-aided design (TCAD) simulations was conducted to evaluate the effects of these parameters on the performance of HJDs. The results indicate that adjusting the hole concentration and expanding the diameter dimensions of the CuO_x_ layer contribute to a lower R_on,sp_ and a higher V_B_. Moreover, CuO_x_/Ga_2_O_3_ HJDs featuring a double-layer CuO_x_ structure were fabricated and optimized. This double-layer CuO_x_ structure design achieved a great balance between the V_B_ and R_on,sp_. The optimized double-layer CuO_x_/Ga_2_O_3_ HJDs exhibited a high V_B_ of 2780 V and a low R_on,sp_ of 6.46 mΩ·cm^2^, further resulting in a power figure of merit (PFOM) of 1.2 GW/cm^2^. These findings suggest that the optimized p-type CuO_x_ layers offer an effective strategy for achieving high-performance Ga_2_O_3_-based bipolar device in power electronics applications.

## 2. Device Structure and Fabrication Process

To investigate the hole concentration in Cu_2_O films, a series of Cu_2_O films were deposited on sapphire substrates using radio frequency (RF) magnetron sputtering technique. The sapphire substrates were pre-cleaned by acetone, isopropyl alcohol, and deionized water to remove surface impurities and organic residues. The target material is high-purity Cu_2_O (99.99%). The RF sputtering power is 100 W, and the sputtering time sets at 30 min. The gas flow rate of Ar is 50 sccm, and the flow rate of O_2_ is varied from 0 sccm to 4 sccm to modulate the hole concentration in the CuO_x_ films. The deposition conditions and results are summarized in Table 1. In addition, an untreated sapphire sheet served as a control sample for comparison. The hole concentration and mobility of the CuO_x_ films were measured using an HL5500PC resistor Hall Effect Measurement System. The surface morphology and root mean square (RMS) surface roughness were detected by Atomic Force Microscope (AFM, MFP-3D SA, Asylum Research, Santa Barbara, CA, USA). Qualitative analysis of CuO_x_ films using Raman spectroscopy (LabRAM HR Evolution, HORIBA, Kyoto, Japan).

The Ga_2_O_3_ epitaxial wafer used in this study was purchased from Novel Crystal Technology, Inc, Japan. It features a 10 μm Si-doped n-type drift layer and 650 μm Sn-doped bulk substrate. Mesa isolation was performed using inductively coupled plasma (ICP) etching with BCl_3_ as the etchant and followed by a 10 min piranha solution treatment (H_2_SO_4_:H_2_O_2_ = 4:1) to remove surface impurities. The depth of the mesa isolation is 650 nm. The distance between the mesa edge and the anode or p^−^ CuO_x_ layer is 5 μm. A Ti/Au (20/100 nm) metal stack was deposited on the backside of the wafer by e-beam evaporation and annealed at 510 °C for 1 min in an N_2_ ambient to create the backside ohmic contacts. Then, the double-layer CuO_x_ films were then sputtered onto the Ga_2_O_3_ drift layer, followed by a lift-off process at room temperature. Finally, a Ni/Au (50/50 nm) metal stack was subsequently deposited on the CuO_x_ layer. Moreover, all the Ga_2_O_3_ HJDs were fabricated with the identical anode metal (radius = 50 μm) to ensure the consistent forward current. In this study, the room temperature forward current–voltage and breakdown voltage measurements were conducted using a Keithley-4200-SCS (TEKTRONIX, INC., Beaverton, ON, USA) and Agilent B1505A (Keysight Technologies, Santa Rosa, CA, USA), respectively.

## 3. Results and Discussion

### 3.1. Characterization of CuO_x_ Films

Table 1 summarizes the deposition parameters for CuO_x_ films, which were deposited for 30 min under varying deposition conditions. The thickness of the CuO_x_ films initially shows an increase but then subsequently decreases, suggesting that an appropriate quantity of O_2_ content can boost the deposition rate of the film, while an excessive amount of O_2_ can suppress it. The hole concentration in the CuO_x_ films rises with increased O_2_ content, likely due to a reduction in the Cu^+^/Cu^2+^ ratio. Since CuO has a lower formation energy for Cu vacancies compared to that of Cu_2_O, it exhibits a higher intrinsic carrier density than Cu_2_O [21]. This result implies that adjusting the Cu^+^/Cu^2+^ ratio could be an effective approach to modulating the hole concentration in CuO_x_ films. The mobility significantly decreases with the increase in hole concentration, primarily due to the enhanced ionized impurity scattering and electron-hole scattering at a high hole concentration [22].

**Figure 1:**
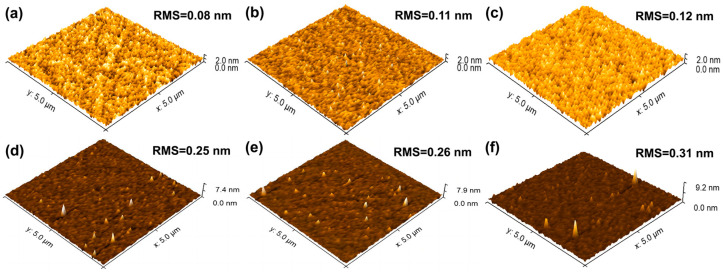
AFM images of (**a**) bare sapphire, (**b**) 50 sccm Ar CuO_x_ film, (**c**) 50 sccm Ar and 1 sccm O_2_ CuO_x_ film, (**d**) 50 sccm Ar and 2 sccm O_2_ CuO_x_ film, (**e**) 50 sccm Ar and 3 sccm O_2_ CuO_x_ film, and (**f**) 50 sccm Ar and 4 sccm O_2_ CuO_x_ film.

The morphology of the CuO_x_ films was measured by an AFM over a 5 × 5 μm^2^ scanning area. Figure 1 compares the three-dimensional surface morphology of the CuO_x_ films deposited under varying O_2_ fluxes. Figure 1a shows the bare sapphire substrate with a root mean square (RMS) surface roughness of 0.08 nm. The RMS values for films in Figure 1b,c are 0.11 nm and 0.12 nm, respectively, indicating that the introduction of a small amount of O_2_ can enhance the deposition rate of CuO_x_ films without significantly impacting their surface morphology. As the O_2_ content continuous to increase, however, the deposition rate of CuO_x_ film gradually declines. It is evident that excessive O_2_ can impede film growth. Additionally, the surface roughness of CuO_x_ films increases notably with higher O_2_ fluxes, with RMS values in Figure 1d–f of 0.25 nm, 0.26 nm, and 0.31 nm, respectively. These results highlight that while a controlled quantity of O_2_ can be beneficial, excessive O_2_ adversely affects both the deposition rate and surface smoothness of the films.

**Figure 2:**
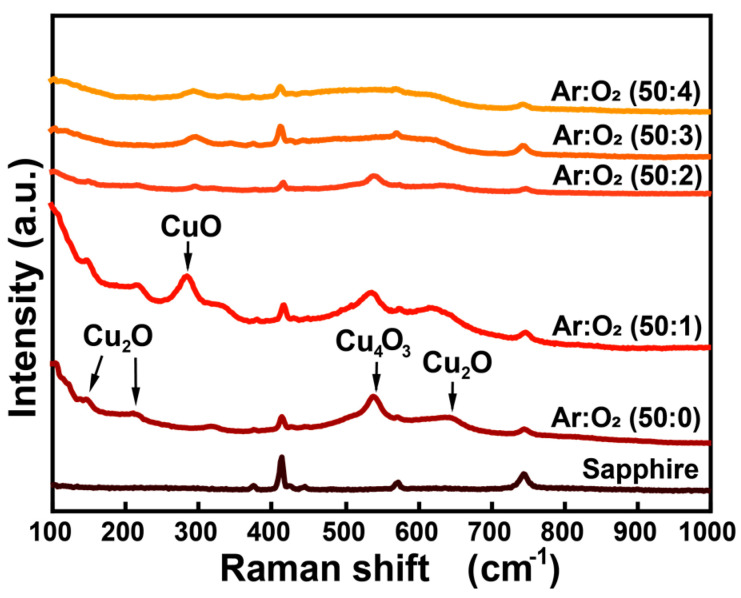
Raman spectra of the samples sputtering under various conditions.

Figure 2 depicts the Raman spectra of various CuO_x_ films sputtered on sapphire substrates, with the spectrum of the bare sapphire substrate serving as a reference. At Ar/O_2_ ratio of 50:0 and 50:1, the characteristic peaks were observed at 150 cm^−1^, 210 cm^−1^, and 630 cm^−1^, corresponding to the Cu_2_O. Additionally, the peak at 300 cm^−1^, characteristic of CuO, indicates that Cu_2_O is gradually oxidized to CuO as O_2_ flow increases. Meanwhile, the peak at 540 cm^−1^ is attributed to the Cu_4_O_3_, a phase containing copper in both +1 and +2 valence states [23]. As the O_2_ introduced increases, the disappearance of the Cu_4_O_3_ peak suggests that the films are nearly completely oxidized to CuO [24]. These results demonstrate that samples with Ar/O_2_ ratios of 50:0 and 50:1 contain a mixture of Cu_2_O, CuO, and Cu_4_O_3_, whereas the samples with higher oxygen content consist predominantly of CuO. These findings align with the AFM analysis, which showed changes in surface morphology with the increasing O_2_ content.

### 3.2. Ga_2_O_3_ HJDs with a Single-Layer CuO_x_ Structure

**Figure 3:**
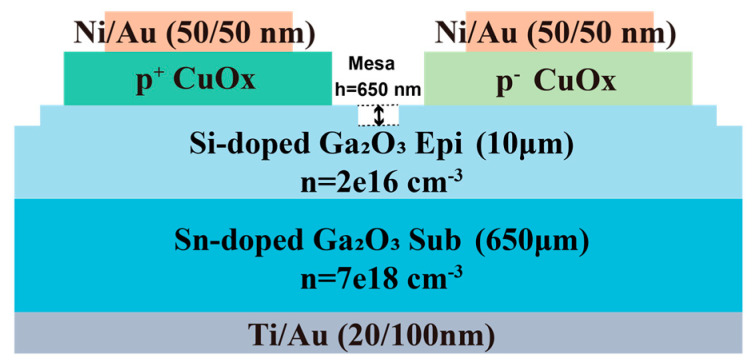
Cross-sectional schematic of the CuO_x_/Ga_2_O_3_ HJDs with a single-layer CuO_x_ structure.

**Figure 4:**
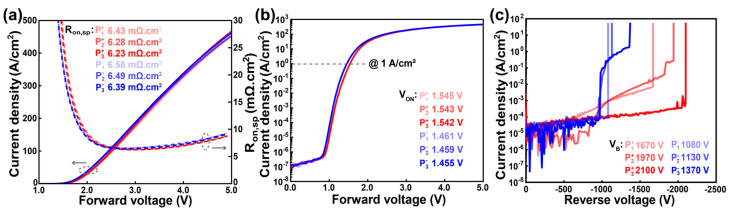
HJDs with a single-layer CuO_x_ structure for (**a**) Linear plots of *I–V* characteristic and extracted R_on,sp_ vs. forward bias. (**b**) Semi-log plots of *I–V* characteristic. (**c**) Reverse *I–V* characteristic.

In this section, we would mainly discuss the effects of the hole concentrations and geometric dimensions of the CuO_x_ layer on the performance of HJDs. Figure 3 shows various vertical CuO_x_/Ga_2_O_3_ HJDs with a single-layer CuO_x_ structure on the same wafer. Table 2 summarizes the key parameters for these Ga_2_O_3_ HJDs with single-layer CuO_x_. The p^+^ CuO_x_ layer had a high hole concentration of 3.9 × 10^19^ cm^−3^ (Ar/O_2_ = 50:3), contrasting with the p^−^ CuO_x_ O layer, which has a lower hole concentration of 3.7 × 10^17^ cm^−3^ (Ar/O_2_ = 50:1). Figure 4a illustrates the forward characteristics of all single-layer p^+^ and p^−^ CuO_x_ HJDs with various dimensions. The ideal factor of p^+^ and p^−^ CuO_x_ HJDs are around 1.8 and 1.6, respectively, indicating that Shockley–Read–Hall (SRH) recombination may be the predominant mechanism. Due to the higher hole concentration and improved electrical conductivity, the HJDs with the p^+^ CuO_x_ layer demonstrate lower R_on,sp_ than those with the p^−^ CuO_x_ layer. Additionally, as the diameter dimensions of the CuO_x_ layer increase, the R_on,sp_ tends to decrease. This is attributed to the larger junction area that facilitates carrier transport [25].

Figure 4b depicts the semi-log plots of the forward *I–V* characteristics, indicating that HJDs with p^+^ CuO_x_ layers have a higher turn-on voltage (V_ON_) than those with p^−^ CuO_x_ layers. The increased hole concentration in the CuO_x_ layer narrows the depletion region, resulting in a stronger built-in electric field and requiring a higher external voltage to initiate conduction, thereby increasing the V_ON_ [26]. The reverse *I–V* characteristics in Figure 4c further reveal that HJDs with p^+^ CuO_x_ layers have a significantly higher V_B_ compared to those with p^−^ CuO_x_ layers. Furthermore, the V_B_ value of HJDs increases with the size of the CuO_x_ layer. The highest V_B_ observed in the single-layer HJD, featuring a p^+^ CuO_x_ layer with 80 μm radius, achieves a PFOM value of 0.71 GW/cm^2^.

**Figure 5:**
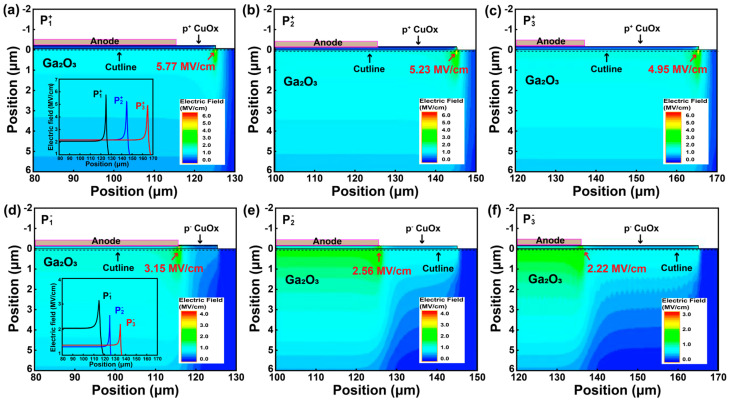
(**a**–**c**) Electric field distributions for HJDs with a single-layer p^+^ CuO_x_ structure. (**d**–**f**) Electric field distributions for HJDs with a single-layer p^−^ CuO_x_ structure.

To comprehensively investigate the impact of geometric dimensions in the p^+^ and p^−^ CuO_x_ layers on the V_B_ performance of HJDs, we analyzed the simulated electric field distributions for single-layer HJDs with varying CuO_x_ layer diameter dimensions under a reverse bias voltage of 1000 V, as shown in Figure 5. The extracted electric field profiles of the HJDs with p^+^ and p^−^ CuO_x_ layers along the cutline at the Ga_2_O_3_ surface are summarized in Figure 5a,d, respectively. In the HJD with p^−^ CuO_x_, the peak electric field is observed beneath the edge of the anode foot, whereas in the HJD with p^+^ CuO_x_, it shifts to the edge of the CuO_x_ foot. Moreover, the HJDs with p^+^ CuO_x_ layers present a significantly higher peak electric field compared to those with p^−^ CuO_x_ layers. This is attributed to the narrowing of the PN junction depletion region as the hole concentration increases, resulting in a stronger electric field intensity concentrated within the junction region [27]. These findings also suggest that optimizing the electric field distribution in the CuO_x_/Ga_2_O_3_ HJDs can be effectively achieved by tuning the hole concentration in the CuO_x_ layer. Furthermore, the size of the CuO_x_ also has an effect on the electric field distribution. As the CuO_x_ layer size increases, the peak electric field within the HJD decreases, leading to a notable enhancement in the V_B_. This improvement is due to the expansion of the PN junction area, which enlarges the depletion region under reverse bias conditions.

### 3.3. Ga_2_O_3_ HJDs with a Double-Layer CuO_x_ Structure

**Figure 6:**
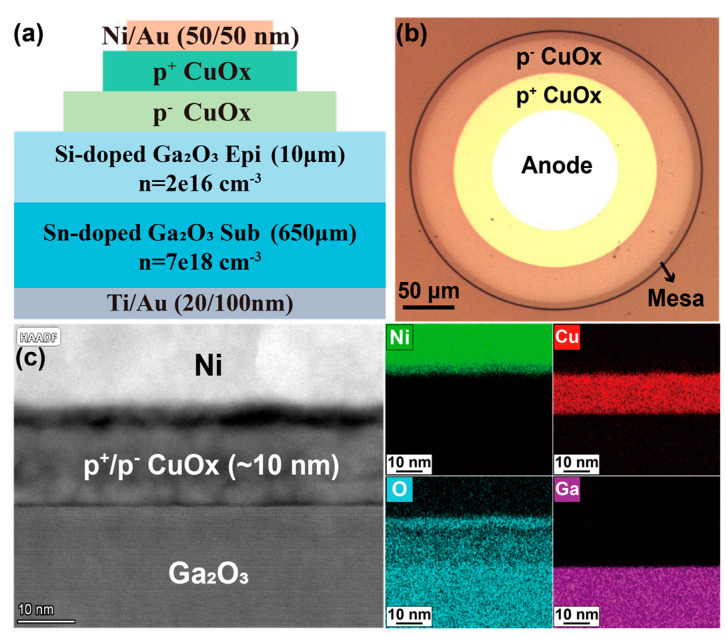
(**a**) Cross-sectional schematic of the CuO_x_/Ga_2_O_3_ HJDs with a double-layer CuO_x_ structure. (**b**) Optical image of the double-layer CuO_x_/Ga_2_O_3_ HJD. (**c**) TEM image and EDS analysis of the double-layer CuO_x_/Ga_2_O_3_ HJD.

To fully optimize the p^+^ and p^−^ CuO_x_ layers, we fabricated the CuO_x_/Ga_2_O_3_ HJDs featuring a double-layer CuO_x_. This section focuses on the impact of the geometric dimensions in the bilayer CuO_x_ on the performance of the HJDs via a comparative analysis of devices fabricated on the samples D_1_–D_5_. Table 3 lists the geometric parameters for these Ga_2_O_3_ HJDs with a double-layer CuO_x_. The radial dimension of the p^+^ CuO_x_ layer was fixed at 80 μm, while the radii of the p^−^ CuO_x_ layer in the samples D_1_–D_5_ were set to 85, 90, 100, 110, and 120 μm, respectively. Figure 6a,b show the device structure and top view of the optical image of the CuO_x_/Ga_2_O_3_ HJD (D_4_). Figure 6c presents the TEM image and EDS analysis of HJD with a double-layer CuO_x_ structure, revealing the polycrystalline nature of the sputtered CuO_x_. It also highlights the sharp and well-defined heterojunction interface, demonstrating the high-quality formation of the CuO_x_/Ga_2_O_3_ HJDs [28].

**Figure 7:**
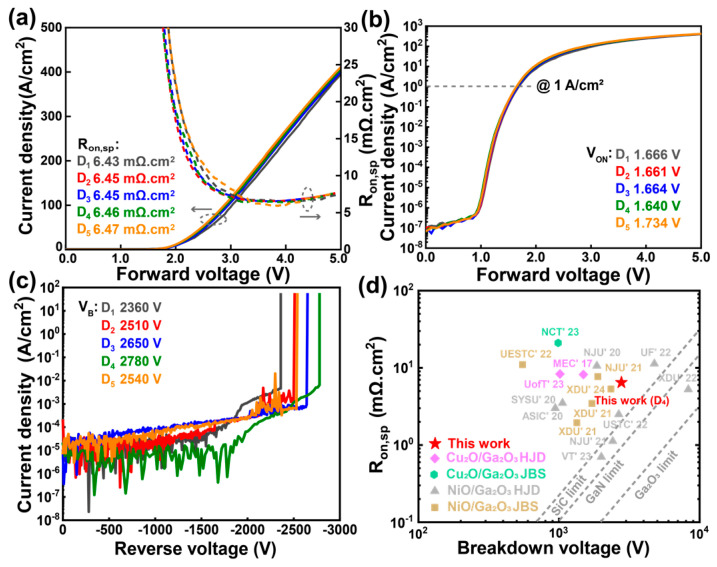
(**a**) Linear plots of *I–V* characteristic and extracted R_on,sp_ vs. forward bias. (**b**) Semi-log plots of *I–V* characteristic. (**c**) Breakdown characteristic for HJDs of D_1_–D_5_. (**d**) Benchmark of R_on,sp_ vs. V_B_ of state-of-the-art Ga_2_O_3_ diodes.

Figure 7a presents the forward linear-scale *I–V* characteristics and the extracted differential R_on,sp_ of the Ga_2_O_3_ HJDs. With increasing the diameter dimensions of the p^−^ CuO_x_ layer size, the R_on,sp_ value of the double-layer HJDs increases slightly from 6.43 mΩ·cm^2^ to 6.47 mΩ·cm^2^, indicating that the expanded p^−^ CuO_x_ layer has minimal impact on the R_on,sp_ and suggesting negligible current diffusion through the bottom p^−^ CuO_x_ layer. The ideal factors of the double-layer HJDs are around 1.7 to 1.9. Figure 7b shows the log-scale *I–V* characteristics, showing a similar V_ON_ across all the devices at a forward current density of 1 A/cm^2^. All the devices demonstrated high rectification ratios exceeding 10^8^. Figure 7c illustrates the measured V_B_ for the double-layer HJDs. The V_B_ increased from 2360 V in D_1_ to 2780 V in D_4_ with a larger p^−^ CuO_x_ layer. This improvement in the V_B_ is mainly attributed to the enhanced depletion depth of the PN junction achieved by the double-layer CuO_x_ structure with varying hole concentrations [27]. This design effectively suppresses the peak electric field at the anode edge and redistributes it within the devices. However, sample D_5_ showed a decreased V_B_ of 2540 V. It indicates that a moderate size distance between the p^+^ and p^−^ CuO_x_ layers is beneficial for the enhancement of V_B_, while an excessive distance (over 30 μm) negatively impacts performance. Figure 7d presents the benchmark plots of the R_on,sp_ versus V_B_ for state-of-the-art Ga_2_O_3_ diodes. The fabricated Ga_2_O_3_ HJDs with a double-layer CuO_x_ structure (D_4_) achieves a PFOM of 1.20 GW/cm^2^. The performance of this HJD is comparable to that of the extensively studied NiO/Ga_2_O_3_ HJDs, demonstrating its competitive potential for high-performance power electronics applications [29,30].

**Figure 8:**
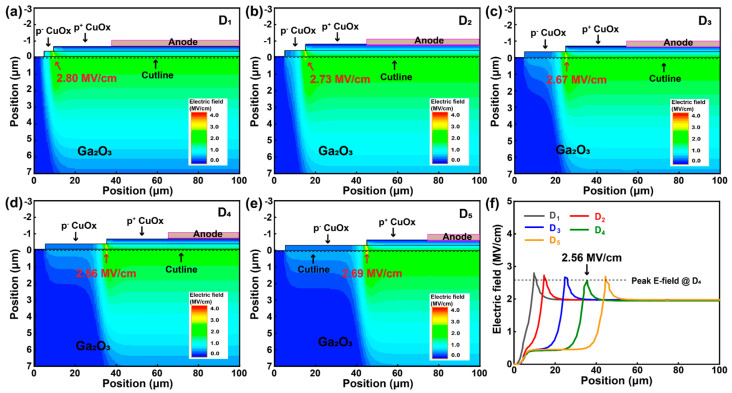
(**a**–**e**) Electric field distribution for HJDs of D_1_–D_5_. (**f**) Extracted electric field profiles along the cutline at the Ga_2_O_3_ surface.

Figure 8a–e show electric field distribution for double-layer HJDs at reverse voltage of 1000 V, while Figure 8f presents the extracted electric field profiles along the cutline at the Ga_2_O_3_ surface. Notably, as the radius difference between the p^+^ and p^−^ CuO_x_ layers increased from 5 to 40 μm, the peak electric field of the double-layer HJDs gradually reduced from 2.80 MV/cm to 2.56 MV/cm. These results are consistent with the V_B_ results discussed previously. Table 4 summarizes the values of the R_on,sp_ and V_B_ for the Ga_2_O_3_ HJDs with a double-layer CuO_x_ structure, along with the calculated PFOM values. The data clearly demonstrate the influence of the p^−^ CuO_x_ layer’s structural parameters on the electrical performance of CuO_x_/Ga_2_O_3_ HJDs. As the radius difference between the p^+^ and p^−^ CuO_x_ layers reached 30 μm, the PFOM value achieved 1.2 GW/cm^2^, representing the highest reported value for CuO_x_/Ga_2_O_3_ HJDs.

## 4. Conclusions

In conclusion, we successfully prepared high-quality CuO_x_ layers and investigated the effects of their geometric dimensions and hole concentration on the electrical characteristics of the CuO_x_/Ga_2_O_3_ HJDs via both experimental analysis and TCAD simulations. These results indicated that tuning the hole concentration and expanding the diameter dimensions of the CuO_x_ layer effectively lowered the R_on,sp_ and raised the V_B_. Moreover, the high-performance CuO_x_/Ga_2_O_3_ HJDs with a double-layer CuO_x_ structure were demonstrated by optimizing these CuO_x_ parameters. It exhibits a high V_B_ of 2780 V and a low R_on,sp_ of 6.46 mΩ·cm^2^, further yielding a record PFOM value of 1.2 GW/cm^2^. This work provides an effective strategy for improving the performance of Ga_2_O_3_-based bipolar power electrons.

## Figures and Tables

**Table 1:** The results of CuO_x_ films deposited under various sputtering conditions.

Power (W)	Ar (sccm)	O_2_ (sccm)	Hole Concentration (/cm^3^)	Mobility (cm^2^/V·s)	Thickness (nm)
100	50	0	1.8 × 10^16^	3.29	85.02
		1	3.7 × 10^17^	0.94	89.68
		2	2.7 × 10^19^	0.28	76.70
		3	3.9 × 10^19^	0.12	68.94
		4	1.4 × 10^20^	0.05	60.09

**Table 2:** Device parameters for the Ga_2_O_3_ HJDs with a single-layer CuO_x_ structure.

Sample ID	CuO_x_ Structure	Hole Concentration (/cm^3^)	Radius of p^+^ CuO_x_ (μm)	Radius of p^−^ CuO_x_ (μm)
$p_{1}^{+}$	single-layer	3.9 × 10^19^	60	/
$p_{2}^{+}$	single-layer	3.9 × 10^19^	70	/
$p_{3}^{+}$	single-layer	3.9 × 10^19^	80	/
$p_{1}^{-}$	single-layer	3.7 × 10^17^	/	60
$p_{2}^{-}$	single-layer	3.7 × 10^17^	/	70
$p_{3}^{-}$	single-layer	3.7 × 10^17^	/	80

**Table 3:** Device parameters for the Ga_2_O_3_ HJDs with a double-layer CuO_x_ structure.

Sample ID	CuO_x_ Structure	Hole Concentration (/cm^3^)	Radius of p^+^ CuO_x_ (μm)	Radius of p^−^ CuO_x_ (μm)
D_1_	double-layer	p^+^/p^−^ (~e^19^/~e^17^)	80	85
D_2_	double-layer	p^+^/p^−^ (~e^19^/~e^17^)	80	90
D_3_	double-layer	p^+^/p^−^ (~e^19^/~e^17^)	80	100
D_4_	double-layer	p^+^/p^−^ (~e^19^/~e^17^)	80	110
D_5_	double-layer	p^+^/p^−^ (~e^19^/~e^17^)	80	120

**Table 4:** Values of the R_on,sp_ and V_B_ for the Ga_2_O_3_ HJDs with a double-layer CuO_x_ structure.

Sample ID	R_on,sp_ (mΩ·cm^2^)	V_B_ (V)	PFOM (GW/cm^2^)
D_1_	6.43	2360	0.87
D_2_	6.45	2510	0.98
D_3_	6.45	2650	1.09
D_4_	6.46	2780	1.20
D_5_	6.47	2540	1.00

## Data Availability

The data that support the findings of this study are available from the corresponding authors upon reasonable request.
